# Change in Cartilage Status of Medial Compartment after Open-Wedge High Tibial Osteotomy without Cartilage Regeneration Procedure: Second Look Arthroscopic Assessment

**DOI:** 10.3390/biomedicines11061639

**Published:** 2023-06-05

**Authors:** Sung-Sahn Lee, Juyong Oh, Dae-Hee Lee

**Affiliations:** 1 Department of Orthopaedic Surgery, Ilsan Paik Hospital, Inje University School of Medicine, Goyangsi 10380, Republic of Korea; sungsahnlee@gmail.com; 2 Department of Orthopaedic Surgery, Samsung Medical Center, Sungkyunkwan University School of Medicine, Seoul 06351, Republic of Korea; dhwndyd007@naver.com

**Keywords:** cartilage, regeneration, high tibial osteotomy, osteotomy

## Abstract

This study investigated the rate of cartilage regeneration after an open-wedge high tibial osteotomy (HTO) without cartilage regeneration by second-look arthroscopy. This study included patients who underwent an open-wedge HTO between July 2014 and March 2019. A total of 65 patients were enrolled. Pre- and postoperative (second-look arthroscopy) hip–knee–ankle (HKA) angle and tibial slope were measured. All patients underwent arthroscopic examination prior to osteotomy. Medial femoral condyle (MFC) and medial tibial plateau (MTP) articular cartilage were evaluated according to the International Cartilage Repair Society (ICRS) grading system. After 26.5 months, second-look arthroscopy was performed with plate removal to identify the cartilage status of the MFC and MTP. The preoperative HKA angle (6.4° ± 2.7°) was well corrected postoperatively (−2.7° ± 2.7°, *p* < 0.001). In terms of MFC on second-look arthroscopy, 29 patients (44.6%) showed an improved ICRS grade, 31 patients (47.7%) were maintained, and 5 patients (7.7%) showed a worse ICRS grade since the prior operation. In the MTP group, 19 patients (29.2%) improved, 44 patients (67.7%) were maintained, and 2 patients (3.1%) worsened. Approximately 44.6% and 29.2% of patients showed improved cartilage statuses on the MFC and MTP after open-wedge HTO without any cartilage regeneration procedures. Cartilage regenerations in both the MFC and MTP did not influence clinical outcomes.

## 1. Introduction

Osteoarthritis (OA) affects the entire synovial joint, including the synovium, cartilage, and subchondral bone. The reaction mechanism of the chondrocyte in OA is represented as (1) proliferation and cell death, (2) changes in synthetic activity and degradation, and (3) formation of osteophytes [1]. The degradation of the cartilage matrix is observed initially in the cartilage superficial zone; however, it extended to deeper as OA advanced. Later, the subchondral bone is also marked as changed, including the increment of cortical plate thickness, change in bone mass of subchondral trabecular bone mass, and alteration of bone turnover [2]. Knee joint OA is also caused by the failed repair of joint damage from stresses. The symptoms of knee OA are represented as pain, decreased joint motion, stiffness, and reduced physical activity. Factors that known to associated with increased risk of knee OA include female sex, older age, obesity, previous knee injury, and varus or valgus malalignment of the knee joint [3].

High tibial osteotomy (HTO) is a surgical method performed in medial compartment osteoarthritis within the varus knee in relatively young patients [4]. In medial compartment osteoarthritis, the mechanical axis is usually medially deviated due to varus deformity, which leads to degenerative changes in the cartilage. HTO is a good surgical option for patients whose mechanical axis is medially deviated, and several studies have shown excellent clinical outcomes after HTO [5,6]. Initially, closed-wedge HTO was widely performed. With the advent of the T-shape locking plate, open-wedge HTO, which provides the advantages including easier surgical techniques and less risk of neurovascular injury, gained popularity among orthopedic surgeons [7].

Numerous anatomical changes could be found following open-wedge HTO. For example, the medial compartment cartilage could be regenerated, the cartilage status of patellofemoral joint or lateral compartment might be degraded, and the alignment of the ankle joint might change [8,9,10]. Among these changes, there is a change in the alignment of the lower extremities from varus to valgus, which leads to a reduction in medial compartment stress. Based on this theoretical basis, remodeling of the medial compartmental articular cartilage could occur after HTO [11,12]. Previous studies reported that 55–92% of patients showed cartilage regeneration in the medial femoral condyle (MFC) or medial tibial plateau (MTP) following HTO [13]. Given the wide range of reported rates of cartilage regeneration in the existing literature, it is crucial to obtain a second-look arthroscopic assessment of cartilage regeneration following open-wedge HTO without additional cartilage regeneration procedures. Moreover, a few studies were focused on the clinical effects of cartilage regeneration after HTO.

The purpose of the present study was (1) to investigate the rate of cartilage regeneration after open-wedge HTO without cartilage regeneration by second-look arthroscopy and (2) to identify the correlation between cartilage regeneration and patients’ reported outcomes. It was hypothesized that approximately one-third of patients would show improvements in cartilage statuses in the medial compartment.

## 2. Materials and Methods

### 2.1. Patients

This retrospective study included patients who underwent open-wedge HTO between July 2014 and March 2019. The inclusion criteria were patients who (1) underwent open-wedge HTO for medial compartment osteoarthritis, (2) did not undergo additional femoral osteotomy or ligament reconstruction, and (3) underwent plate removal surgery with second-look arthroscopy. Exclusion criteria were as follows: (1) history of previous knee surgery, (2) systemic or traumatic arthritis, or (3) ligament insufficiency. The surgical indications for open-wedge HTO were (1) symptomatic medial compartment osteoarthritis (Kellgren–Lawrence grade ≥ 1) or MFC osteonecrosis, (2) varus malalignment of 5° to 15°, (3) flexion contracture <15°, and (4) absence of osteoarthritis of the lateral compartment. Demographic data including age, sex, body mass index, and affected side was obtained. This study was approved by the Ethics Committee of our institution (SMC2023-04-113), and written informed consent was obtained from all patients.

### 2.2. Surgical Procedure and Rehabilitation

All surgical procedures were performed by a senior surgeon (D-H.L.). All patients underwent arthroscopic examination prior to the osteotomy. Meniscectomy was performed when a flap tear of the medial meniscus was present. A longitudinal tear of more than 1 cm or a root tear of the medial meniscus was repaired. MFC and MTP articular cartilage was evaluated. The status of the articular cartilage was graded according to the International Cartilage Repair Society (ICRS) grading system as follows: grade 1, superficial lesions and superficial fissures and cracks; grade 2, lesions extending the cartilage depth <50%; grade 3, lesions extending the cartilage depth >50% but not involving the subchondral bone; and grade 4, the subchondral bone involved [14]. Cartilage regeneration procedures including microfracture or subchondral drilling were not performed in all patients. The preoperative amount of correction was measured using the miniaci method on whole-leg standing radiographs with the patella facing forward [15]. An anteromedial longitudinal skin incision was made from the superomedial aspect of the inferior patellar pole, 4–5 cm below the tibial tuberosity. The pes anserine tendon was released from the tibia. The superficial medial collateral ligament was transected when performing oblique osteotomy. Under intraoperative fluoroscopy, two guidewires were inserted in the plane of the osteotomy site, and osteotomy was performed with an oscillating saw for a distance of up to 1 cm from the lateral cortex. The vertical osteotomy part of the biplane osteotomy was performed approximately 1 cm behind the tibial tubercle to avoid injury to the bony attachment of the patellar tendon. During osteotomy under intraoperative fluoroscopy, the mechanical axis passed through 62.5% of the medial border of the tibial plateau. The osteotomy site was stabilized using a fixed-angle plate with interlocking screws (TomoFixTM; Synthes, Bettlach, Switzerland) after achieving the target osteotomy width. An allogeneic bone chip graft was then inserted into the osteotomy gap.

Patients were instructed to start isometric quadriceps, active ankle, and straight-leg-raising exercises on the day after surgery. Tolerable range of motion was allowed 2 days after surgery. Partial weight bearing was permitted in the first week, and full weight bearing was allowed 6 weeks after surgery.

### 2.3. Second-Look Arthroscopic Evaluation

Second-look arthroscopy was performed with plate removal. Plate removal was performed at least 18 months later after open-wedge HTO surgery if patients wanted removal surgery after the explanation of advantages and disadvantages. MFC and MTP articular cartilage was also evaluated using the ICRS grading system. The patients were divided according to ICRS grade on MFC or MTP. The regeneration groups on MFC or MTP were defined as those with downgraded ICRS grades on MFC or MTP. The non-regeneration group were defined as those with same or upgraded ICRS grades. Furthermore, the status of the regenerated cartilage was evaluated according to the modified staging system of Kanamiya et al. with second-look arthroscopy [11]. The conventional grading system was classified as grade 1 to 4: grade 1: no regenerative change, grade 2: white scattering with fibrocartilage, grade 3: partial coverage with fibrocartilage, and grade 4: even coverage with fibrocartilage. A modified grading system was added with grades 0 and 5: grade 0: degenerative change, and grade 5: over-coverage with fibrocartilage.

### 2.4. Clinical and Radiographic Evaluation

Clinical outcomes, including Western Ontario and McMaster University Osteoarthritis (WOMAC) index [16] and International Knee Documentation Committee (IKDC) subjective score [17], were investigated preoperatively and in the second-look arthroscopy. Preoperative and postoperative clinical outcomes were also compared between the groups. Clinical outcomes were compared between the regeneration and non-regeneration group.

Preoperative plain radiographs, including standing anteroposterior view and Rosenberg view, were analyzed to determine Kellgren–Lawrence grade [18]. The radiographs were obtained before surgery and in second-look arthroscopy, including whole leg standing radiographs and lateral view radiographs. The hip–knee–ankle (HKA) angle was defined as the angle between the lines drawn (1) from the center of the femoral head to the center of the knee and (2) drawn from the center of the ankle to the center of the knee on the whole standing leg radiographs. The negative and positive HKA angles were defined valgus and varus, respectively [19,20]. The medial proximal tibial angle (MPTA) was measured as the angle between the tibial mechanical axis and the articular surface of the proximal tibia on whole leg standing radiographs [21,22,23]. Tibial slope was defined as the angle between the midline of the tibia shaft and the line of posterior inclination of the tibial plateau on lateral view radiographs [24]. All measurements were repeated 6 weeks later for intra-observer reliability assessment. Intraclass correlation coefficients (ICCs) were performed for inter- and intra-observer reliability assessments.

### 2.5. Statistical Analysis

Statistical analysis was performed by SPSS software (version 27, IBM, Chicago, IL, USA). The Shapiro–Wilk test was performed to evaluate the normality of distribution. A paired *t*-test was used to compare preoperative and postoperative (second-look arthroscopy) radiographic measurements. The chi-squared test was used for categorical variables to compare the cartilage grade between the prior operation and the second-look examination. The Student’s *t*-test was used to compare clinical outcomes between regeneration and non-regeneration groups. Uni- and multivariable logistic regression was used to analyze the factors that affect regeneration of cartilage. Independent variables were demographic data, pre- and postoperative HKA angle, MPTA, tibial slope and preoperative ICRS grade of MFC and MTP. Statistical significance was set at *p* < 0.05. Totals of 29 and 36 patients were allocated to the regeneration group and non-regeneration group on MFC. A 16.1-point difference in the WOMAC index was reported to be clinically important [6]. It would take a 93.1% statistical power to detect a difference of at least 16.1 points with a standard deviation of 20 points in the WOMAC index (α = 0.05).

## 3. Results

A total of 65 patients was enrolled in the current study. The demographic data are shown in Table 1. The mean period from prior surgery to second-look examination was 26.5 ± 9.1 months.

All inter- and intra-observer ICCs showed good agreement with respect to the reliability of radiographic measurements (>0.80). The preoperative HKA angle (6.4° ± 2.7°) was well corrected postoperatively (−2.7° ± 2.7°, *p* < 0.001). The tibial slope was well maintained (preoperative vs. postoperative, 80.6° ± 3.5° vs. 80.4° ± 3.0°, *p* = 0.58). Clinical outcomes, including WOMAC index and IKDC subjective score, were significantly improved postoperatively (Table 2).

The cartilage status of the MFC and MTP according to the ICRS grade is presented in Table 3. In terms of MFC on second-look arthroscopy, 29 patients (44.6%) showed an improved ICRS grade, 31 patients (47.7%) were maintained, and 5 patients (7.7%) showed a worse ICRS grade since the prior operation. With respect to MTP, 19 patients (29.2%) were improved, 44 patients (67.7%) were maintained, and 2 patients (3.1%) were worse in second-look arthroscopy. The detailed pre- and postoperative ICRS grades of patients who showed improvements are represented in Table 4. In terms of comparing the ICRS grading change between genders, there was no statistical difference (Table 5).

**Figure 1:**
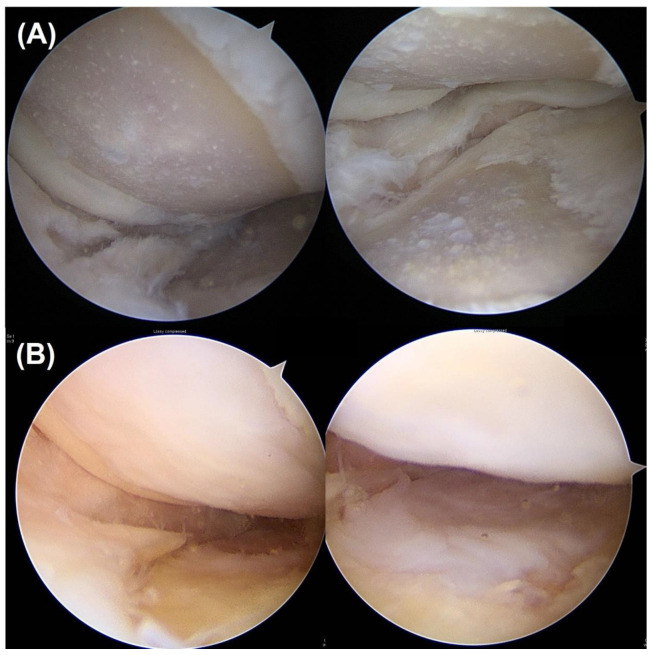
(**A**) Fifty-nine-year-old female had ICRS grade 4 chondral lesions at both the MFC and MTP at prior HTO surgery. (**B**) After 25 months, a second-look examination was performed. Both MFC and MTP cartilage showed even coverage with ICRS grade 1. ICRS, International Cartilage Repair Society; MFC, medial femoral condyle; high tibial osteotomy; MTP, medial tibial plateau.

**Figure 2:**
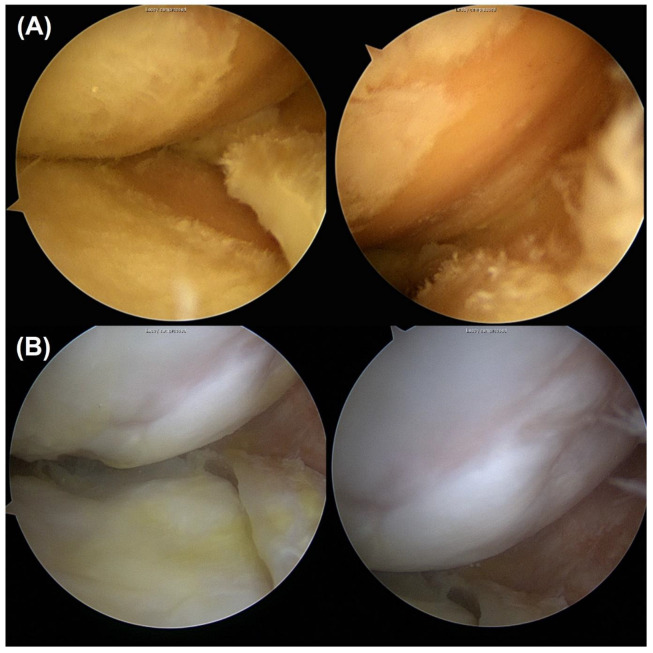
(**A**) Sixty-year-old male had ICRS grade 4 chondral lesions at both MFC and MTP at prior HTO surgery. (**B**) Twenty-three months later, he underwent a second-look examination. Both MFC and MTP cartilage showed over-coverage. ICRS, International Cartilage Repair Society; MFC, medial femoral condyle; MTP, medial tibial plateau; high tibial osteotomy.

Among the patients who showed improved MFC cartilage status compared to prior surgery, 21 patients showed partial improvement, 5 patients showed full improvement, and 3 patients showed over-coverage. In terms of MTP, 16, 1, and 2 patients showed partial, full, and over improvement, respectively (Table 6, Figure 1 and Figure 2).

In terms of comparing the clinical outcomes between the regeneration and non-regeneration groups on the MFC, pre- and post-operative WOMAC indexes and IKDC subjective scores were not statistically different (Table 7). These were also not statistically different between the regeneration and non-regeneration groups on the MTP (Table 8).

In respect of the predictive factor for the regeneration of the MFC, the lesser postoperative HKA angle (more valgus) was the only associated factor (OR 0.5, 95% CI 0.3–0.8, *p* = 0.01, Table 9). On the other hand, no factor was statistically associated with MTP cartilage regeneration on logistic regression analysis (Table 10).

## 4. Discussion

The principal finding of our study was that 44.6% and 29.2% of patients showed improved cartilage statuses on the MFC and MTP after open-wedge HTO without any cartilage regeneration procedures. However, cartilage regeneration did not influence clinical outcomes.

The physiological tibiofemoral joint load distribution was not consistent. The load of the medial compartment was more than 60% of the joint load because of the varus alignment [25]. Therefore, the medial compartment was commonly degraded in the early stage of osteoarthritis. As the osteoarthritis in the medial compartment progressed, the degradation of cartilage in the medial compartment increased, and the severity of the varus malalignment intensified, establishing a self-perpetuating cycle. HTO is an effective surgical option for medial compartmental osteoarthritis. The main principle of HTO is to correct varus lower extremity alignment; the pressure should be transferred from the medial compartment to lateral compartment, which is relatively healthy. Two different HTO techniques are commonly used and performed: a lateral closed-wedge HTO and a medial open-wedge HTO [7]. Traditionally, the open-wedge HTO has gradually taken the place of the closed-wedge HTO, although closed-wedge HTOs were more commonly performed in the past. The advantages of an open-wedge HTO over a closed-wedge HTO include easier control of the degree of correction, less extensive soft tissue dissection, sparing of the proximal tibiofibular joint or the non-necessity of the fibular osteotomy, and the avoidance of serious complications such as peroneal nerve palsy [26]. Numerous studies have concentrated on comparing the two different surgical techniques in terms of tibial slope change, patellar height, survival rate, and complications [7,25,27,28]. The complication rate and survival rate were not different, but open-wedge HTO was associated with increased tibial slope, leg length, and joint line elevation [19,29]. Open-wedge HTO has been reported to be beneficial in patients with medial compartmental degeneration, contributing from chronic posterior cruciate ligament insufficiency because it increases the posterior tibial slope as a result of the unique anatomic configuration of the proximal tibia [30]. However, theoretically, an increased tibial slope is an associated adverse effect on anterior cruciate ligament [31]. We think that postoperative changes in slope may have an impact on cartilage regeneration by influencing the integrity and function of the cruciate ligaments. Therefore, it is important to preserve the postoperative slope for outcomes. In the current study, statistical significance was not observed in the tibial slope, pre- and post-surgery. Hence, it can be inferred that the alteration in slope had minimal impact on cartilage regeneration in our study. A comparative investigation evaluating the extent of cartilage regeneration between closed-wedge HTO and open-wedge HTO is deemed a valuable avenue for future research.

Knee osteoarthritis is a common chronic joint disease and a major cause of pain and functional limitations. Multifactorial factors are associated with knee joint cartilage degeneration [32]. Increased joint load and loss of shock absorption could contribute for the progression of knee joint arthritis [33]. HTO could correct the abnormal biomechanical forces, thereby allowing the medial compartment of the knee joint to be offloaded [6]. Interestingly, it was shown that cartilage could be regenerated by reducing the medial compartmental load alone without cartilage regeneration, including cartilage debridement, drilling, or microfractures [11,33,34,35]. Some studies have investigated regeneration after HTO using second-look arthroscopy, but the results varied. Kanamiya et al. [11] investigated cartilage regeneration in the medial compartment after closed-wedge HTO. A total of 58 patients was enrolled in their study, and the mean period from initial osteotomy surgery to second-look arthroscopy was 18 months. In their study, approximately 55% of knees showed partial or even coverage with cartilage, 34% showed white scattering of fibrocartilage, and 11% showed no repair. Wakabayashi et al. [35] evaluated 37 cases of eburnation cartilage lesions and 36 cases of fibrillation cartilage lesions on initial arthroscopy. All patients underwent closed-wedge HTO, and second-look arthroscopy was postoperatively performed after 1 year. Among the patients who had grade 4 lesions, 62% and 63% showed improved cartilage statuses on MFC and MTP, respectively. In contrast, in patients who had grade 3 lesions, 9% showed improved cartilage statuses on both MFC and MTP. They concluded that fibrillated cartilage had little potential for cartilage repair after closed-wedge HTO without cartilage procedures. Koshino et al. [36] also performed closed-wedge HTO and second-look arthroscopy 2 years later. A total of 146 knees was enrolled; 32% showed full coverage with regenerated cartilage, and 68% showed no or partial regeneration. Jung et al. [34] performed a second-look examination after open-wedge HTO. A total of 159 patients was enrolled in their study, and second-look arthroscopy was performed 2 years after surgery. More than grade 2 regeneration (white scattering, partial coverage, or even coverage) was achieved on the MFC in 92% of knees and on the MTP in 69% of knees. Kim et al. [33] also evaluated patients who underwent open-wedge HTO and second-look arthroscopy after 25 months. Among 104 knees, 52% and 35% showed improved cartilage on MFC and MTP, respectively. Otsuki et al. [37] demonstrated that 58% (82 of 142 patients) showed more than partial regeneration of the medial compartmental cartilage on second-look arthroscopy after open-wedge HTO, although the ratio of cartilage regeneration varied among previous studies because the method of evaluating cartilage differed from author to author. However, it seems clear that cartilage was regenerated at a certain rate after HTO without cartilage regeneration procedures.

The main goal of a HTO is to improve patient function and relieve pain [8]. Undoubtedly, HTO has been revealed to enhance postoperative outcomes in patients with medial compartment osteoarthritis with varus deformity [38]. Numerous investigations aiming to identify the factors that contribute to favorable patient-reported outcomes following HTO have been actively pursued [6,39,40,41]. The correlation between postoperative lower extremity alignment and postoperative outcome has been steadily reported. Kettelkamp et al. [42] reported favorable results in cases with a femorotibial angle (FTA) of 157°. As open-wedge HTO has been widely performed, and whole-leg standing radiographs have been used for analysis, a postoperative HKA angle of 2–7° valgus or a weight-bearing line ratio of 57–67% was considered an ideal target after surgery [39,43]. Lee et al. [6] compared the patients’ reported outcomes, including IKDC subjective score, Knee Injury and Osteoarthritis Outcome Score, and Kujala score between under-correction, appropriate correction, and over-correction. They concluded that over-corrected surgery (weight-bearing line raito > 67%) was correlated with inferior patients’ reported outcomes. Traditionally, younger age is known to be associated with successful outcomes after HTO [38]. On the other hand, an opposite opinion was suggested. Kohn et al. [40] had investigated the influence of age on clinical outcomes after HTO. They had generated 13 pairs of patients with a mean age at operation of 57 versus 42 (15 years younger). The patients were matched by gender, operation/osteosynthesis method, body mass index, and follow-up period. The patients were compared in respect of Lysholm score, Tengner score, and visual analog pain scale. All outcomes were not significantly different between both groups. They concluded that HTO is an effective surgery for medial compartment osteoarthritis, independent of the patient’s age. Two different studies investigated the effect of preoperative radiographic kissing lesions on postoperative clinical outcomes. Kim et al. [38] compared the American knee society (AKS) knee and function scores between patients with radiologic kissing lesions (17 patients) and non-kissing lesions (105 patients). Patients with kissing lesions showed similar improvements of AKS knee scores (11.9 ± 5.4 vs. 10.6 ± 3.9, *p* = 0.348) and function scores (21.2 ± 3.3 vs. 21.6 ± 4.2, *p* = 0.679) with non-kissing lesions. Shon et al. [41] also compared the Hospital for Special Surgery score, WOMAC index, and Tegner score between kissing lesions (21 cases) and non-kissing lesions (22 cases). All patients’ reported outcomes were not significantly different. In the current study, cartilage regeneration was not associated with superior patients’ outcomes. It seems that further research is needed on the clinical significance of cartilage regeneration.

Jung et al. [34] conducted a comparison of the regeneration rate of medial compartmental cartilage between postoperative HKA angles greater than 0° and less than 0° following open-wedge HTO. The group with a postoperative HKA angle greater than 0° (under-corrected group) exhibited a significantly lower rate of cartilage regeneration compared to the group with a postoperative HKA angle less than 0° (appropriate or over-corrected group). Our results aligned with this previous study, showing that a greater degree of valgus correction is associated with regeneration of MFC cartilage. Furthermore, we found that Kim et al. [33] reported a relationship between lower BMI and cartilage regeneration of MFC and MTP following open-wedge HTO. These findings indicate that the factors influencing cartilage regeneration have yet to be clearly identified. Therefore, we believe that a larger volume study is necessary to identify more accurate predictive factors of cartilage regeneration.

Our study had several limitations. First, our study was a retrospective, nonrandomized, sequential review. Therefore, selection bias could have occurred. Second, the follow-up period was relatively short. Third, histological analysis was not performed; therefore, it was difficult to determine the histology of the regenerated cartilage. Fourth, the size of the cartilage defect was not considered.

## 5. Conclusions

Approximately 44.6% and 29.2% of patients showed improved cartilage statuses on the MFC and MTP after open-wedge HTO without any cartilage regeneration procedures. Cartilage regenerations in both the MFC and MTP did not influence clinical outcomes.

## Figures and Tables

**Table 1:** Demographic data of the patients (mean ± standard deviation).

Demographic Characteristics	
Sample size (number)	65
Sex (male/female)	21/44
Age (years)	58.0 ± 9.0
Body mass index (kg/m^2^)	27.5 ± 3.1
Affected side (right/left)	24/41
Kellgren–Lawrence grade (1/2/3/4)	9/14/24/18
Second look period (months)	26.5 ± 9.1
Additional procedure (number)	37
Partial meniscectomy	17
Meniscal repair (root repair)	20(15)

**Table 2:** Comparison of clinical and radiographic outcomes between pre-operation and post-operation. Values are mean ± standard deviation.

	Preoperation	Postoperation	*p* Value
WOMAC index	61.1 ± 11.8	22.2 ± 8.4	<0.001
IKDC subjective score	25.3 ± 10.3	58.2 ± 17.3	<0.001
HKA angle, °	6.4 ± 2.7	−2.7 ± 2.7	<0.001
Tibial slope, °	80.6 ± 3.5	80.4 ± 3.0	0.58
MPTA, °	84.3 ± 10	93.1 ± 2.9	<0.001

WOMAC, Western Ontario and McMaster University Osteoarthritis; IKDC, International Knee Documentation Committee; HKA, hip–knee–ankle; MPTA, medial proximal tibial angle.

**Table 3:** Cartilage status of medial femoral condyle and medial tibial plateau according to ICRS (International Cartilage Repair Society) grading system at prior surgery and second-look arthroscopy.

	Prior Operation		Second Look	
ICRS Grade	MFC	MTP	MFC	MTP
I	3	1	7	1
II	7	13	12	19
III	16	16	26	25
IV	39	35	20	20

MFC, medial femoral condyle; MTP, medial tibial plateau.

**Table 4:** Pre- and postoperative ICRS (International Cartilage Repair Society) grading of medial femoral condyle (MFC) and medial tibial plateau (MTP) among the patients who showed improvement.

		**Postoperative MFC ICRS Grade**		
		**1**	**2**	**3**
Preoperative MFC ICRS grade	1	0	0	0
	2	2	0	0
	3	1	3	0
	4	1	5	17
		**Postoperative MTP ICRS Grade**		
		**1**	**2**	**3**
Preoperative MTP ICRS grade	1	0	0	0
	2	0	0	0
	3	0	3	0
	4	0	4	12

**Table 5:** Comparison of ICRS grading change between male and female.

		Sex		*p* Value
		Male	Female	
Medial femoral condyle	Improvement	10 (47.6%)	19 (43.2%)	0.794
	No improvement	11 (52.4%)	25 (56.8%)	
Medial tibial plateau	Improvement	5 (23.8%)	14 (31.8%)	0.572
	No improvement	16 (76.2%)	30 (68.2%)	

**Table 6:** Modified staging system of Kanamiya et al. [11] in second-look arthroscopy compared to prior surgery.

	MFC	MTP
Grade 0: Degenerative change	5	2
Grade 1: No regenerative change	31	44
Grade 2: White scattering with fibrocartilage	8	7
Grade 3: Partial coverage with fibrocartilage	13	9
Grade 4: Even coverage with fibrocartilage	5	1
Grade 5: Over coverage with fibrocartilage	3	2
Total:	65	65

MFC, medial femoral condyle; MTP, medial tibial plateau.

**Table 7:** Comparison of pre- and postoperative clinical outcomes between regeneration and non-regeneration groups on medial femoral condyle. Values are mean ± standard deviation.

	Regeneration on MFC (*n* = 29)	Non-Regeneration on MFC (*n* = 36)	*p* Value
Preoperative WOMAC index	60.2 ± 11.5	61.9 ± 12.2	0.633
Preoperative IKDC subjective score	26.6 ± 11.5	24.1 ± 9.1	0.43
Postoperative WOMAC index	23.2 ± 9.1	21.2 ± 7.9	0.424
Postoperative IKDC subjective score	57.5 ± 18.5	58.8 ± 16.4	0.796

WOMAC, Western Ontario and McMaster University Osteoarthritis; IKDC, International Knee Documentation Committee.

**Table 8:** Comparison of pre- and postoperative clinical outcomes between regeneration and non-regeneration groups on medial tibial plateau. Values are mean ± standard deviation.

	Regeneration on MTP (*n* = 19)	Non-Regeneration on MTP (*n* = 46)	*p* Value
Preoperative WOMAC index	58.6 ± 12.0	62.5 ± 11.6	0.286
Preoperative IKDC subjective score	27.5 ± 12.3	24.0 ± 8.9	0.289
Postoperative WOMAC index	23.6 ± 8.5	21.4 ± 8.5	0.411
Postoperative IKDC subjective score	61.5 ± 21.4	56.2 ± 14.5	0.386

WOMAC, Western Ontario and McMaster University Osteoarthritis; IKDC, International Knee Documentation Committee.

**Table 9:** Logistic regression analysis of the medial femoral condyle cartilage regeneration.

Variables	Univariable Analysis				Multivariable Analysis			
	Adjusted OR	95% CI		*p* Value	Adjusted OR	95% CI		*p* Value
		Lower	Upper			Lower	Upper	
Age	0.9	0.6	1.3	0.518				
Sex	0.3	0.1	1.3	0.253				
BMI	1.1	0.6	2.1	0.758				
Preoperative HKA angle	1.4	0.7	3	0.372				
Postoperative HKA angle	0.4	0.2	0.8	0.018	0.5	0.3	0.8	0.01
Preoperative MPTA	0.8	0.5	1.4	0.439				
Postoperative MPTA	1.5	0.6	3.7	0.399				
Preoperative tibial slope	2.2	0.8	6.3	0.131				
Postoperative tibial slope	0.5	0.2	1.1	0.095				
Preoperative ICRS grade of MFC	1.5	0.5	8.1	0.153				
Preoperative ICRS grade of MTP	0.2	0.1	5.7	0.281				

HKA, hip–knee–ankle; MPTA, medial proximal tibial angle; ICRS, International Cartilage Repair Society.

**Table 10:** Logistic regression analysis of the medial tibial plateau cartilage regeneration.

Variables	Adjusted OR	95% CI		*p* Value
		Lower	Upper	
Age	0.9	0.7	1.1	0.186
Sex	1.3	0.3	4.8	0.174
BMI	1.1	0.8	1.6	0.542
Preoperative HKA angle	1.1	0.7	1.6	0.665
Postoperative HKA angle	0.8	0.5	1.3	0.393
Preoperative MPTA	0.8	0.6	1.2	0.307
Postoperative MPTA	0.9	0.6	1.4	0.674
Preoperative tibial slope	1	0.7	1.4	0.98
Postoperative tibial slope	1	0.7	1.5	0.79
Preoperative ICRS grade of MFC	1.3	0.3	5.9	0.757
Preoperative ICRS grade of MTP	2.1	0.5	8.3	0.17

HKA, hip–knee–ankle; MPTA, medial proximal tibial angle; ICRS, International Cartilage Repair Society.

## Data Availability

Not applicable.
